# Integrative Taxonomy of *Polynema* (*Doriclytus*) (Hymenoptera: Mymaridae) from Oriental China: Three New Species and Five New Records Revealed by Morphological and Molecular Analyses †

**DOI:** 10.3390/insects16111166

**Published:** 2025-11-15

**Authors:** Yanyan Liu, Serguei V. Triapitsyn, Dan Zhang, Jinling Wang, Zhulidezi Aishan

**Affiliations:** 1 College of Life Science and Technology, Xinjiang University, Urumqi 830017, China; 2 Xinjiang Key Laboratory of Biological Resources and Genetic Engineering, Urumqi 830017, China; 3 Entomology Research Museum, Department of Entomology, University of California, Riverside, CA 92521, USA; 4 Characteristic Laboratory of Forensic Science in Universities of Shandong Province, Shandong University of Political Science and Law, Jinan 250014, China

**Keywords:** Mymaridae, fairyfly, morphology, DNA barcoding, species delimitation, new species, new records, Oriental region, China

## Abstract

*Polynema* Haliday, 1833 (Hymenoptera: Chalcidoidea: Mymaridae), one of the most species-rich genera in the family, comprises egg parasitoids with diverse hosts across multiple insect orders, some serving as biological control agents for agricultural and forestry pests. The subgenus *Polynema* (*Doriclytus* Foerster, 1847), characterized by pronounced morphological conservatism, has historical taxonomic challenges due to reliance on external morphological characteristics. This study employed an integrative taxonomic approach, combining morphological and molecular analyses, to investigate *P.* (*Doriclytus*) diversity in the Oriental region of China. Eight species were identified, including three new species—*P.* (*Doriclytus*) *acutum* Wang & Aishan, sp. nov., *P.* (*Doriclytus*) *daliense* Wang & Aishan, sp. nov., and *P.* (*Doriclytus*) *longicornia* Wang & Aishan, sp. nov.—and five species newly recorded from China: *P.* (*Doriclytus*) *alalatum* Rehmat & Anis, 2016, *P.* (*Doriclytus*) *bicolorigastra* Rehmat & Anis, 2016, *P.* (*Doriclytus*) *dhenkunde* Mani & Saraswat, 1973, *P.* (*Doriclytus*) *dunense* Hayat & Anis, 1999, and *P.* (*Doriclytus*) *tyakshiense* Irfan & Anis, 2023. Comprehensive morphological descriptions and diagnostic illustrations are provided for all new taxa, with key diagnostic features detailed for the newly recorded species. Molecular analysis of COI sequences using both the Assemble Species by Automatic Partitioning (ASAP) and Generalized Mixed Yule Coalescent (GMYC) models yielded congruent species delimitation results, with genetic distances between delimited species showing maximum intraspecific divergence of 1.51% and interspecific divergences of 3–12% within the 470 bp COI barcode region. The deposition of 32 novel COI sequences in GenBank significantly enhances molecular resources for Mymaridae systematics.

## Simple Summary

Minute parasitoids of the genus *Polynema* play a potentially significant role in controlling pests that harm crops and forests by laying their eggs inside the eggs of their hosts. However, identifying these parasitoids is tricky because they look very similar, and their taxonomy is in flux. Our study tackled this problem by combining close-up examinations of the parasitoids’ morphological features with DNA analysis to separate the species. We aimed to discover and describe new parasitoid species in China’s Oriental region. Eight species were identified, including three new ones named *Polynema* (*Doriclytus*) *acutum* Wang & Aishan, *Polynema* (*Doriclytus*) *daliense* Wang & Aishan, *Polynema* (*Doriclytus*) *longicornia* Wang & Aishan., and five previously described ones being recorded in China for the first time. By studying their DNA, we confirmed these are distinct species, with genetic differences ranging from about 3% to about 12% within the 470 bp COI barcode region. We also added 32 new COI sequences to the global database, thus helping future research.

## 1. Introduction

The genus *Polynema* Haliday, 1833 (Hymenoptera: Chalcidoidea: Mymaridae) is one of the largest genera in the family, with 224 described species distributed across all zoogeographical regions prior to this study [1]. *Polynema* exhibits a remarkably diverse host range, serving as egg parasitoids of agricultural and forestry pests spanning at least six insect orders: Hemiptera, Diptera, Odonata, Coleoptera, Lepidoptera, and Thysanoptera [2,3]; however, the published records from Diptera, Coleoptera, Lepidoptera, and Thysanoptera eggs are doubtful and thus need confirmation. Within this diverse genus, the subgenus *Polynema* (*Doriclytus* Foerster, 1847) represents a morphologically distinct and ecologically important group. Originally established as a genus by Foerster in 1847 with *Doriclytus vitripennis* Foerster as the type species, *Doriclytus* was subsequently redefined as a subgenus of *Polynema* by Triapitsyn & Fidalgo [4,5]. Some species within *P.* (*Doriclytus*) are known biological control agents, morphologically distinguished by three diagnostic characteristics: (1) a pit adjacent to each torulus on the face; (2) propleura meeting anteriorly along the midline and thus the prosternum closed; and (3) male genitalia with digiti lacking hooks, occasionally bearing minute denticles [4].

Despite extensive taxonomic research, species delineation in *P.* (*Doriclytus*) has primarily relied on external morphological characters [6,7,8,9,10,11,12,13]. The minute body size and conserved morphological features among these parasitoids often hinder accurate species identification, particularly when distinguishing closely related species. This challenge is especially pronounced in the Oriental region, where the subgenus remains poorly documented despite the region’s high biodiversity and the presence of numerous potential host species sometimes requiring biological control. Species in the Oriental region have not been described, and their distribution remains unknown. The complex topography and diverse climates of Oriental China likely harbor substantial undiscovered *P.* (*Doriclytus*) diversity, warranting comprehensive taxonomic investigation. To address these limitations, the integration of DNA barcoding [14] with morphological data has emerged as a powerful approach for species delimitation in Mymaridae. Successful applications include demonstration that several nominal *Anagrus* Haliday, 1833 species (*A. erythroneurae* S. Trjapitzin & Chiappini, 1994, *A. lindberginae* Nugnes & Viggiani, 2014, etc.) are conspecific with *A. atomus* (Linnaeus, 1767) across Europe and North America [15], and characterization of *Pseudanaphes yadongicus* Aishan & Cao, 2023 from Xizang, China [16].

However, traditional threshold-based DNA barcoding often struggles to distinguish intra- and interspecific variation in *P.* (*Doriclytus*) due to overlapping genetic distances and limited diagnostic power in closely related species. To achieve robust and comprehensive species delimitation, this study uses morphology and molecular genetic data to identify the following integrative taxonomy framework. 

In this study, we employ an integrative taxonomic approach to investigate the diversity of *P.* (*Doriclytus*) in the Oriental region of China. We describe three new species of *P.* (*Doriclytus*) and report five known species as new records from this region. By generating COI sequence data and applying molecular species delimitation, we provide convergent morphological and molecular evidence for species boundaries, enhancing understanding of the subgenus’ diversity and distribution and contributing to the broader application of integrative taxonomy in minute parasitoid wasps.

## 2. Materials and Methods

### 2.1. Specimen Collection

Fresh specimens of Chalcidoidea were collected using sweep nets and Malaise traps throughout the Oriental region of China during field surveys conducted between 2020 and 2023. Upon arrival at the laboratory facilities of Xinjiang University in Urumqi, Xinjiang Uyghur Autonomous Region of China, specimens belonging to *Polynema* (*Doriclytus*) were sorted from bulk chalcidoid and immediately preserved in 99% ethanol, then stored at −20 °C for subsequent morphological examinations and molecular analyses. Additional examined material comprised slide-mounted specimens from the collection of Fujian Agriculture and Forestry University in Fuzhou, Fujian, China (FAFU).

Type specimens of newly described species and DNA sequencing voucher specimens were deposited in the Insect Collection of the College of Life Science and Technology, Xinjiang University, Urumqi, China (ICXU).

### 2.2. Morphological Study and Imaging

Preliminary morphospecies identifications were conducted by Serguei V. Triapitsyn and Zhulidezi Aishan based on detailed morphological examinations. Ethanol-preserved specimens were photographed prior to destructive sampling using a Nikon SMZ25 stereomicroscope (Nikon Corporation, Tokyo, Japan). After DNA extraction, specimens were dissected and permanently slide-mounted in Canada balsam on glass slides under a Jiangnan SE2200 stereomicroscope (Shanghai Shengke Corporation, Shanghai, China).

High-resolution digital images of slide-mounted structures were captured using a Nikon Eclipse Ci-L biological microscope (Nikon Corporation, Tokyo, Japan) equipped with a digital camera system. Morphometric measurements were taken from these images. Morphological terminology follows Triapitsyn [12,17]. Abbreviations used for morphological characters are as follows: F1–F6, funiculars 1–6; MPS, multiporous plate sensilla; T, tarsomere. All measurements are given in micrometers (μm) unless stated otherwise. Image stacks were digitally combined using image-stacking software Helicon Focus v6.7.1 (Helicon Soft Ltd., Kharkiv, Ukraine), and the resulting composite images were processed in Adobe Photoshop 2022 (Adobe Inc., San Jose, CA, USA).

### 2.3. DNA Extraction, PCR Amplification, and Sequencing

Whole genomic DNA was extracted from the whole specimens of 32 individuals using the TIANamp Genomic DNA Kit (TIANGEN Biotech, Beijing, China) following the manufacturer’s protocol. The specimens after DNA extraction were subsequently slide-mounted and retained as a morphological voucher and deposited in ICXU. A partial fragment of the mitochondrial cytochrome c oxidase subunit I (COI) gene was amplified using the forward primer C1-J-1718 (5′-GGAGGATTTGGAAATTGATTAGTTCC-3′) [18] and the reverse primer HCO-2198 (5′-TAAACTTCAGGGTGACCAAAAAATCA-3′) [19]. PCR amplification was performed in 30 μL reactions containing: 15 μL of Premix Taq polymerase (Sangon Biotech, Shanghai, China), 1.5 μL of each primer (10 μM), 5 μL of DNA template, and 7 μL double-distilled water (ddH_2_O).

The thermal cycling profile consisted of an initial denaturation at 94 °C for 3 min; followed by 35 cycles of denaturation at 94 °C for 30 s, annealing at 50 °C for 30 s, and extension at 72 °C for 1 min, with a final extension at 72 °C for 7 min. PCR products were purified and sequenced in both directions by Sangon Biotech, Shanghai, China.

### 2.4. Sequence Processing and Phylogenetic Analysis

Forward and reverse sequences were assembled and edited using BioEdit v7.0.9.0 [20] to generate consensus sequences. Sequence identity was verified through BLASTn v2.9.0 searches against the NCBI database to exclude potential contamination [21]. Verified COI sequences were aligned using the ClustalW [22] algorithm and manually trimmed in BioEdit v7.0.9.0. The final alignment was translated into amino acid sequences in MEGA 11 [23] to check for premature stop codons.

Both distance-based and tree-based species delimitation methods were employed to complement morphological data: the Assemble Species by Automatic Partitioning (ASAP) method, which uses hierarchical clustering of pairwise genetic distances with objective partition scoring [24], and the Generalized Mixed Yule Coalescent (GMYC) model, which leverages phylogenetic trees to identify transitions between interspecific (Yule) and intraspecific (coalescent) branching patterns [25]. The integration of distance-based clustering, phylogenetic coalescent modeling, and morphological assessment ensures a comprehensive evaluation of species limits in this taxonomically challenging group.

Species delimitations were conducted using the following distance-based (ASAP) and tree-based (GMYC) approaches. For ASAP, the verified COI alignment was subjected to SpartExplorer (https://spartexplorer.mnhn.fr/; accessed on 25 July 2025) to conduct ASAP analysis with default settings. Prior to GMYC species delimitation, duplicate haplotypes were removed from the dataset of 32 sequences using BioEdit v7.0.9.0, yielding 23 unique haplotypes for subsequent analysis. An ultrametric phylogenetic tree was constructed for GMYC analysis using Bayesian inference in BEAST v1.10.4 [26]. Substitution model selection was performed using the ModelFinder approach [27] implemented in IQ-TREE v1.6.12 [28]. Based on the Bayesian Information Criterion (BIC), which has been shown to perform well in avoiding overfitting for phylogenetic datasets [29], the K3Pu+F+G4 model was identified as the best-fit. Since this model is unavailable in BEAST, we employed the more general GTR + Γ substitution model, which encompasses K3Pu as a special case (Supplementary Material Figure S1). The analysis incorporated a relaxed uncorrelated lognormal molecular clock [30] and a Yule speciation tree prior, with all other priors set to default. The analysis ran for 50 million generations, sampling every 5000 generations. Convergence and adequate effective sample sizes (ESS > 200) for all parameters were confirmed using Tracer v1.7.2 [31]. After discarding the initial 25% as burn-in, a maximum clade credibility (MCC) tree was generated from the posterior distribution using TreeAnnotator v1.10.4 [32]. Species delimitation hypotheses were evaluated using the single-threshold GMYC model implemented in the ‘splits’ package [25,33] for R v4.3.3 [34]. 

Pairwise genetic distances were calculated using the Kimura 2-parameter (K2P) substitution model in MEGA 11 [23]. A Neighbor-joining (NJ) tree was constructed as a backbone tree to show the above species debilitation results based on K2P distances, with node support assessed using 500 bootstrap replicates. The final NJ tree was visualized and edited using iTOL v6 [35] and Adobe Illustrator 2022 (Adobe Inc., San Jose, CA, USA).

## 3. Results

### 3.1. Species Identification and Delimitation

**Figure 1:**
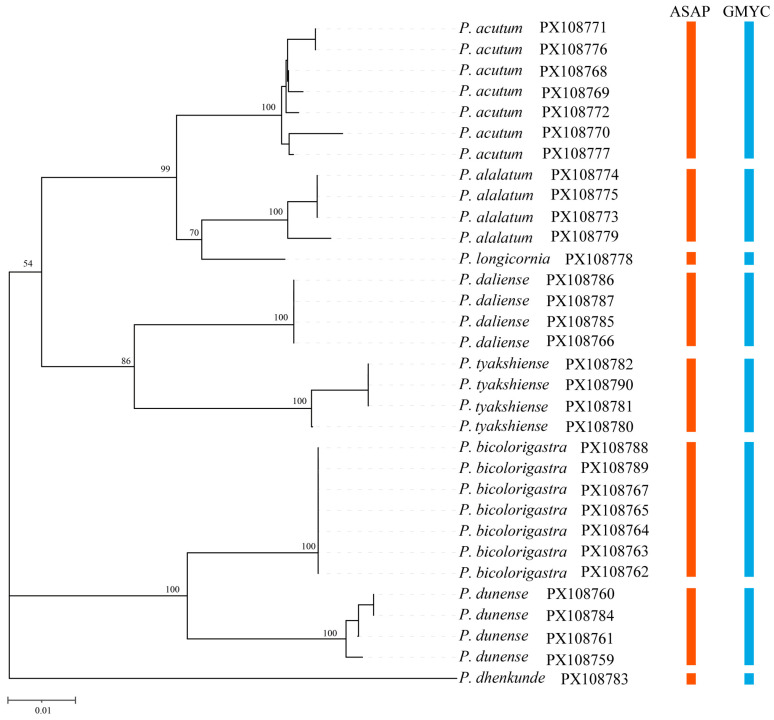
Neighbor-joining tree based on K2P genetic distances calculated from 470 bp COI sequences. Bootstrap support values (≥50%) are shown above interspecific nodes. All terminal species assignments are supported by morphological and molecular genetic species delimitation.

A total of 44 individuals representing eight morphologically identified species of *Polynema* (*Doriclytus*) were examined. Of these, 32 specimens successfully yielded high-quality COI sequences that were used for phylogenetic reconstruction (Figure 1), resulting in a trimmed alignment matrix of 470 bp with no gaps. All newly generated sequences have been deposited in GenBank under accession numbers PX108759-PX108790.

Pairwise genetic distances calculated using the K2P model ranged from 3% to 12% among species and 0 to 1.51% within species (Supplementary Material Table S1). Both the ASAP and single-threshold GMYC analyses delimited eight putative species from the COI dataset, confirming the eight species initially identified based on morphological characters (Figure 1).

### 3.2. Taxonomic Treatment of Polynema (Doriclytus)

#### 3.2.1. Key to Species of *Polynema* (*Doriclytus*) from the Oriental Region of China (Females)

1 Antennal scape with cross ridges…………***P.* (*Doriclytus*) *alalatum* Rehmat & Anis**

- Antennal scape smooth………………...…………………………………………………2

2 Scutellum with frenal foveae……………………..………………………………………3

- Scutellum without frenal foveae…………………………………………………………4

3 Scutellum with a row of foveae; clava relatively narrow and long, at least 3.8× as long as wide; F2 and F3 subequal in length, both the longest funiculars; ovipositor 0.6–0.7× length of metatibia…………………………….… ***P.* (*Doriclytus*) *dunense* Hayat & Anis**

- Scutellum with bits of foveae; clava at most 3.6× as long as wide; F2 the longest funicular, F3 shorter than F2; ovipositor 0.8–1.2× length of metatibia……………………………….. …..***P.* (*Doriclytus*) *bicolorigastra* Rehmat & Anis**

4 Antenna with clava fusiform, apically acute; ovipositor markedly exserted beyond apex of gaster ………………………..***P.* (*Doriclytus*) *acutum* Wang & Aishan sp. nov.**

- Antenna with clava not acute; ovipositor exserted slightly beyond apex of gaster…...5

5 Fore wing relatively narrow, 3.7–4.3× as long as wide; propodeum with a short, inconspicuous median carina……………..***P.* (*Doriclytus*) *dhenkunde* Mani & Saraswat**

- Fore wing relatively wide, at most 3.5 × as long as wide……………………………….6

6 Propodeum with a complete median carina, extending to anterior margin of propodeum…………………………..***P.* (*Doriclytus*) *longicornia* Wang & Aishan sp. nov.**

- Propodeum with median carina incomplete, not extending to anterior margin of propodeum………………………….…………………………………………………………..7

7 Posterior half of petiole with numerous transverse striations; ovipositor 1.2–1.5× as long as metatibia………………….. ***P.* (*Doriclytus*) *daliense* Wang & Aishan sp. nov.**

- Posterior half of petiole smooth; ovipositor 0.9–1.0× length of metatibia…………………………………………***P.* (*Doriclytus*) *tyakshiense* Irfan & Anis**

#### 3.2.2. *Polynema* (*Doriclytus*) Foerster, 1847

*Doriclytus* Foerster, 1847 [5], pp. 226–227. Type species: *Doriclytus vitripennis* Foerster, 1847.

*Polynema* (*Doriclytus*) Foerster: Triapitsyn & Fidalgo, 2006 [4], pp. 57–60 (as a subgenus of *Polynema*).

Diagnosis. Body predominantly dark brown to black. Face with a distinct pit adjacent to each torulus. Propleura contiguous anteriorly along the midline, thus the prosternum closed anteriorly. Male genitalia with digiti lacking hooks, occasionally with minute denticles [4].

#### 3.2.3. *Polynema* (*Doriclytus*) *acutum* Wang & Aishan, sp. nov.

Diagnosis. Female antenna with F1 subequal to F4 in length; F2 the longest funicular; F6 with 1 MPS; clava fusiform, apically acute, 4.0–5.8× as long as wide, longer than the combined length of F4–F6, with 8 MPS. Scutellum without frenal foveae; propodeum with a complete, Y-shaped median carina. Ovipositor long, markedly exserted beyond apex of gaster (by 0.3× its own length). 

**Figure 2:**
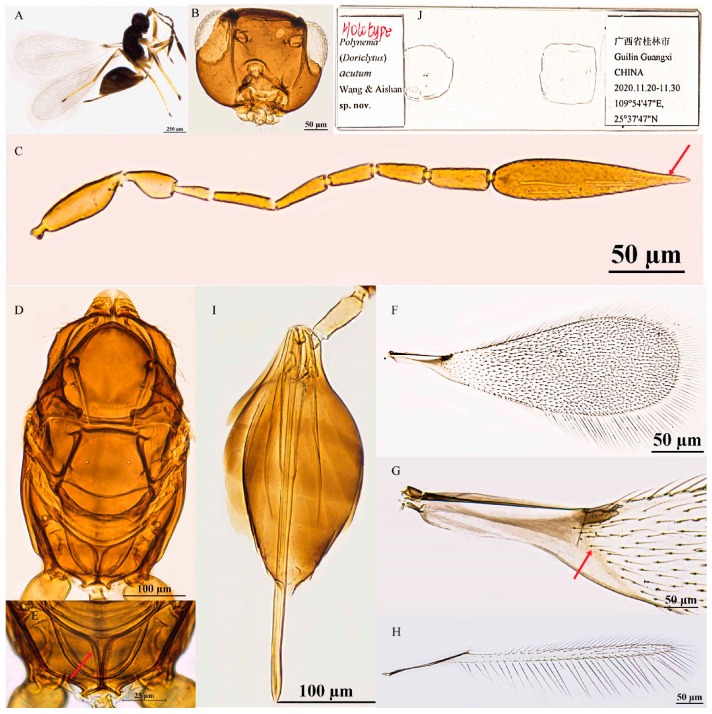
*Polynema* (*Doriclytus*) *acutum* sp. nov. female. (**A**) body; (**B**) head; (**C**) antenna; (**D**) mesosoma in dorsal view; (**E**) propodeum; (**F**,**G**) fore wing; (**H**) hind wing; (**I**) metasoma in dorsal view; (**J**) holotype slide.

Female. Body length 587–652 μm (n = 10; Figure 2A). Body dark brown; antenna brown with scape and pedicel light brown; petiole light yellow; legs light brown, tarsomeres 1–3 yellowish-brown, tarsomere 4 brown.

Head in frontal view 0.6–0.7× as high as wide (Figure 2B). Antenna with scape smooth, 3.7–4.4× as long as wide (including short radicle); pedicel 1.7–2.0× as long as wide; each funicular longer than wide; F1 subequal to F4 in length; F2 equal to F6 in length, both the longest funiculars; F3 and F5 subequal in length, shorter than F2 and F6; F6 with 1 MPS; clava apically acute and fusiform, 4.0–5.8× as long as wide, longer than combined length of F4–F6, with 8 MPS (Figure 2C).

Mesosoma smooth, 1.6–1.7× as long as wide; pronotum medio-longitudinally divided, bearing five setae on each side along the anterior margin; scutellum 0.9–1.0× as long as wide, subequal to mesoscutum in length, without frenal foveae; propodeum with a complete, Y-shaped median carina (Figure 2D,E). Fore wing 3.0–3.2× as long as wide; disk transparent, densely setose beyond venation, discal setae originating at base of stigmal vein; marginal setae longer, longest marginal seta 0.3–0.5× greatest forewing width; marginal vein short, with 1 dorsal macrochaeta, not contacting posterior margin (Figure 2F,G). Hind wing slender and narrow, 21.4–27.0× as long as wide; disk transparent, with two rows of setae; longest marginal seta 3.6–4.3× greatest hind wing width (Figure 2H).

Metasoma longer than mesosoma; petiole 3.0–3.6× as long as wide, with sparse transverse striations. Ovipositor 1.3–1.4× as long as gaster, exserted beyond gastral apex by 0.3× total ovipositor length; ovipositor 1.9–2.1× mesotibia length and 1.5–1.8× metatibia length (Figure 2I).

Holotype measurements (μm) (Figure 2J). Head height/width 94:130; mesosoma length/width 215:131; mesoscutum length/width 76:116; scutellum length/width 70:75; propodeum median carina length 24; petiole length/width 66:21; gaster length/width 277:121; ovipositor length 382, exserted portion 107; antennal segments length/width—scape 73:19, pedicel 36:19, F1 25:7, F2 40:8, F3 35:11, F4 25:12, F5 36:12, F6 40:14, clava 131:24; forewing length/width 694:224, longest marginal seta length 109; hind wing length: width 527:21, longest marginal seta length 89; mesotibia length 185; metatibia length 217.

Male. Unknown.

Etymology. The specific name refers to the acutely pointed clava of the female antenna.

Type material. Holotype ♀: CHINA, Guangxi, Guilin, 25°37′47″ N, 109°54′47″ E, 1100 m, 20–30.XI.2020. Paratypes: CHINA: Guangxi, Guilin, 25°37′47″ N, 109°54′47″ E, 1100 m: 10–20.XI.2020, 1♀; 20–30.XI.2020, 1♀. Hunan, Chenzhou, 24°56′44″ N, 112°54′08″ E, 1108 m, 17–27.XII.2020, 1♀. Jiangxi, Shangrao, 29°06′06″ N, 117°56′42″ E, 1365 m: 10.V.2022, 1♀; 30. IV.2025, 1♀. Zhejiang, Hangzhou, 30°19′50″ N, 119°26′26″ E, 1100 m: 5.XI.2019, 1♀; 28.VIII.2020, 1♀; 10.XI.2020, 1♀; 27.XI.2020, 1♀. Holotype and all the paratypes are slide-mounted and deposited in ICXU.

Hosts. Unknown.

Distribution. Oriental region (CHINA: Guangxi, Hunan, Jiangxi, Zhejiang).

Remarks. This new species is similar to *P.* (*Doriclytus*) *assamense* Hayat & Singh, 2001 from India, but can be distinguished from the latter by the following characters: *P.* (*D.*) *acutum* propodeum with a complete median carina, whereas that of *P*. (*D*.) *assamense* propodeum with median carina incomplete (p. 142, in [8]). Additionally, *P.* (*D*.) *acutum* hind wing 21.4–27.0× as long as wide, whereas that of *P.* (*D*.) *assamense* hind wing 21.0× as long as wide (p. 142, in [8]).

#### 3.2.4. *Polynema* (*Doriclytus*) *daliense* Wang & Aishan, sp. nov.

Diagnosis. Female antenna with F2 the longest funicular; F4 and F6 subequal in length, F6 with 1 MPS; clava 3.1–3.5× as long as wide, equal to the combined length of F4–F6, with 9 MPS. Propodeum with an incomplete median carina, not extending to the anterior margin. Ovipositor slightly exserted beyond apex of gaster (by 0.1–0.2× its own length).

**Figure 3:**
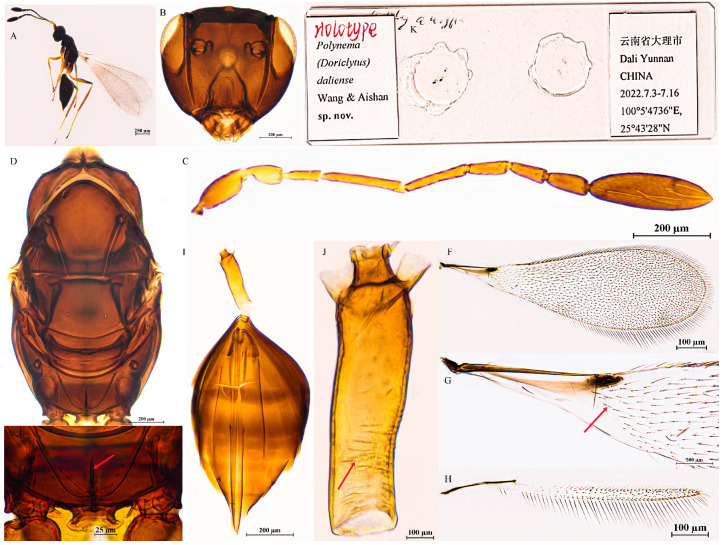
*Polynema* (*Doriclytus*) *daliense* sp. nov. female. (**A**) body; (**B**) head; (**C**) antenna; (**D**) mesosoma in dorsal view; (**E**) propodeum; (**F**,**G**) fore wing; (**H**) hind wing; (**I**) met-asoma in dorsal view; (**J**) petiole; (**K**) holotype slide.

Female. Body length 692–855 μm (n = 4; Figure 3A). Body dark brown; antenna brown, with pedicel yellowish-brown; petiole light brown; legs brown, with tarsomeres 1–3 yellowish-brown.

Head in frontal view 0.5–0.7× as high as wide (Figure 3B). Antenna with scape smooth, 3.0–3.5× as long as wide (including short radicle); pedicel 2.0–2.9× as long as wide; each funicular longer than wide; F1 equal to F5 in length, both the shortest funiculars; F2 the longest funicular; F3 shorter than F2; F4 and F6 subequal in length; F6 with 1 MPS; clava 3.1–3.5× as long as wide, equal to combined length of F4–F6, with 9 MPS (Figure 3C).

Mesosoma smooth, 1.6–1.7× as long as wide; pronotum mediolongitudinally divided, bearing four setae on each side along anterior margin; scutellum lacking a row of frenal foveae; propodeum with median carina incomplete, not extending to anterior margin (Figure 3D,E). Fore wing 3.0–3.3× as long as wide, disk hyaline, densely setose beyond venation, discal setae originating at base of stigmal vein; marginal vein short, with one dorsal macrochaeta, not contacting posterior margin (Figure 3F,G). Hind wing slender and narrow, 21.0–26.8× as long as wide, disk transparent, with two rows of setae; longest marginal seta 2.0–3.6× greatest hind wing width (Figure 3H).

Metasoma distinctly longer than mesosoma; petiole 2.8–4.3× as long as wide, posterior half with numerous transverse striations. Ovipositor slightly exserted beyond gastral apex, 1.0–1.1× as long as gaster, exserted portion 0.08–0.09× ovipositor length; ovipositor 1.5–1.7× mesotibia length and 1.2–1.5× metatibia length (Figure 3I,J).

Holotype measurements (μm) (Figure 3K). Head height/width 110:167; mesosoma length/width 290:172; mesoscutum length/width 109:146; scutellum length/width 88:146; propodeum median carina length 29; petiole length/width 104:26; gaster length/width 350:204; ovipositor length 353, exserted portion 31; antennal segments length/width—scape 70:23, pedicel 41:21, F1 43:11, F2 89:11, F3 70:12, F4 45:15, F5 43:18, F6 45:19, clava 134:39; forewing length/width 875:264, longest marginal seta length 78, discal setae lengths 7–20; hind wing length/width 713:29, longest marginal seta length 93; mesotibia length 231; metatibia length 297.

Male. Unknown.

Etymology. The specific name refers to the type locality.

Type material. Holotype ♀: CHINA, Yunnan, Dali, 25°43′28″ N, 100°54′36″ E, 2924 m, 3–16.VII.2022. Paratypes: CHINA, Yunnan, Dali, 25°43′28″ N, 100°54′36″ E, 2924 m: 3–16.VII.2022, 1♀; 1–16.IX.2022, 1♀; 16.VI–2.VII.2023, Yanqiong Peng, 1♀. Holotype and all the paratypes are slide-mounted and deposited in ICXU.

Hosts. Unknown.

Distribution. Oriental region (CHINA: Yunnan).

Remarks. This species closely resembles *P.* (*Doriclytus*) *tyakshiense* but can be distinguished from the latter by the following characters: scape non-circular in cross-section (vs. circular in *P. tyakshiense*); mesoscutum shorter than scutellum (vs. mesoscutum equal in length to scutellum in *P. tyakshiense*); scutellum lacking a row of frenal foveae (vs. scutellum with a row of frenal foveae in *P. tyakshiense*; p. 66, Figure 8 in [7]).

#### 3.2.5. *Polynema* (*Doriclytus*) *longicornia* Wang & Aishan, sp. nov.

Diagnosis. Female antenna with F1 the shortest funicular; F2 the longest funicular, subequal to F3 in length; F4 subequal to F5 in length; F6 with 1 MPS; clava 3.7× as long as wide, longer than the combined length of F4–F6, with 7 MPS. Propodeum with a complete median carina extending to the anterior margin. Fore wing disk setae extending basally to about mid-length of the submarginal vein. Ovipositor slightly exserted beyond the apex of the gaster (by 0.1× its own length).

**Figure 4:**
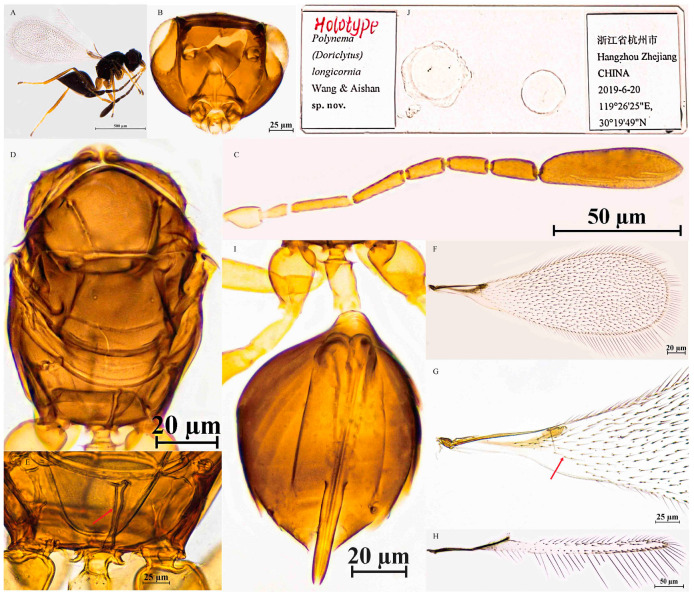
*Polynema* (*Doriclytus*) *longicornia* sp. nov. female. (**A**) body; (**B**) head; (**C**) antenna; (**D**) mesosoma in dorsal view; (**E**) propodeum; (**F**,**G**) fore wing; (**H**) hind wing; (**I**) metasoma in dorsal view; (**J**) holotype slide.

Female. Body length 575 μm (n = 1; Figure 4A). Body black; antenna brown, scape and pedicel yellowish-brown; petiole light yellowish-brown; legs light brown, tarsomeres 1–3 yellowish-brown.

The head in frontal view is 0.6× as high as wide (Figure 4B). Antenna with scape smooth, 4.2× as long as wide (including short radicle); pedicel 1.8× as long as wide, each funicular longer than wide; F1 the shortest funicular; F2 the longest funicular; F3 0.9× as long as F2; F4 subequal to F5 in length; F6 with 1 MPS; clava 3.7× as long as wide, longer than combined length of F4–F6, with 7 MPS (Figure 4C).

Mesosoma smooth, 1.4× as long as wide; pronotum divided mediolongitudinally, bearing 5 setae on each side along anterior margin; propodeum with a complete median carina extending to anterior margin (Figure 4D,E). Fore wing 2.9× as long as wide; disk hyaline, setose beyond venation, extending basally to about mid-length of submarginal vein; longest marginal seta 0.3× greatest width of fore wing; marginal vein short, with 1 dorsal macrochaeta, not contacting posterior margin (Figure 4F,G). Hind wing slender and narrow, 19.9× as long as wide; disk hyaline, with two rows of setae in the middle of the disk; longest marginal seta 3.1× greatest width of hind wing (Figure 4H).

Metasoma longer than mesosoma; petiole 4.4× as long as wide, without striations. Ovipositor subequal to gaster length, slightly exserted beyond gastral apex (by 0.1× ovipositor length); ovipositor 1.6× mesotibia length and 1.3× metatibia length (Figure 4I).

Holotype measurements (μm) (Figure 4J). Head height/width 82:144; mesosoma length/width 194:135; mesoscutum length/width 58:119; scutellum length/width 67:78; propodeum median carina length 35; petiole length/width 79:18; gaster length/width 220:139; ovipositor length 226, exserted portion 29; antennal segments length/width—scape 42:10, pedicel 29:16, F1 21:10, F2 47:10, F3 44:10, F4 32:13, F5 31:13, F6 38:16, clava 115:31; forewing length/width 526:182, longest marginal seta length 58, discal setae length 6–14; hind wing length/width 398:20, longest marginal seta length 62; mesotibia length 142; metatibia length 176.

Male. Unknown.

Etymology. The species name is a noun in apposition referring to the elongate clava of the female antenna.

Type material. Holotype ♀: CHINA, Zhejiang, Linan, 30°19′49″ N, 119°26′25″ E, 1200 m, 20.VI.2019 (slide-mounted, ICXU). 

Hosts. Unknown.

Distribution. Oriental region (CHINA: Zhejiang).

Remarks. This new species is morphologically similar to *P.* (*Doriclytus*) *alalatum* but differs in key antennal and wing characters. The clava of *P.* (*D.*) *longicornia* bears 7 MPS, whereas that of *P.* (*D.*) *alalatum* has 9 MPS (*P.* 158, Figure 2 in [8]). Additionally, the fore wing discal setae of *P.* (*D.*) *longicornia* extend to the mid-length of the submarginal vein in *P.* (*D.*) *longicornia*, in contrast to *P.* (*D.*) *alalatum*, in which they originate at the base of the stigmal vein (p. 158, Figure 3 in [8]).

#### 3.2.6. *Polynema* (*Doriclytus*) *alalatum* Rehmat & Anis, 2016

*Polynema* (*Doriclytus*) *alalatum* Rehmat & Anis, 2016 [8], pp. 140, 143–144.

**Figure 5:**
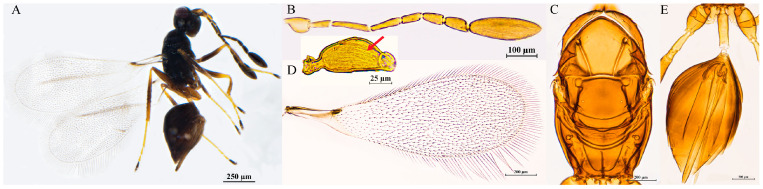
*Polynema* (*Doriclytus*) *alalatum* Rehmat & Anis, 2016, female. (**A**) body; (**B**) antenna; (**C**) mesosoma in dorsal view; (**D**) fore wing; (**E**) metasoma in dorsal view.

Diagnosis. ♀ (♂ unknown). Body length 491–830 μm (n = 5; Figure 5A). Body and antenna dark brown; petiole light brown; legs dark brown with T1–T3 yellowish-brown, T4 brown. Antenna with scape 2.6–2.9× as long as wide, with cross ridges; F2 the longest funicular; F1, F4, F5, and F6 subequal in length; F6 with 1 MPS; clava 2.2–3.1× as long as wide, longer than the combined length of F4–F6, with 8 MPS (Figure 5B). Mesosoma 1.5–1.9× as long as wide; pronotum with 3 setae on each side along anterior margin; scutellum with placoid sensilla centrally positioned, without a row of frenal foveae; propodeum with a complete median carina extending to anterior margin (Figure 5C). Fore wing 2.8–3.1× as long as wide; discal setae originating at base of stigmal vein (Figure 5D). Ovipositor slightly exserted beyond the apex of gaster, 1.0–1.4 × length of metatibia (Figure 5E). 

Material examined. CHINA: Fujian, Quanzhou, 25°40′58″ N, 118°11′05″ E, 1320 m, 5.V.2021, Xiaolei Huang, 1♀. Guangxi, Guilin, 25°37′47″ N, 109°54′48″ E, 1100 m, Xiaolong Lin: 10–20.XI.2020, 1♀; 20–30.XI.2020, 1♀. Hunan, Chenzhou, 24°56′44″ N, 112°54′08″ E, 1108 m, 17–27.XII.2020, 1♀. Xizang, Linzhi, 29°39′10″ N, 94°21′39″ E, 2990 m, 2.IX.2002, Naiquan Lin, 1♀ (FAFU). All specimens listed above are slide-mounted and deposited in ICXU unless otherwise noted.

Distribution. Oriental region (China: Fujian, Guangxi, Hunan, Xizang; India).

Hosts. Unknown.

Remarks. *Polynema* (*Doriclytus*) *alalatum* was originally described by Rehmat and Anis [8] as having the clava with 9 MPS, marginal vein with dorsal macrochaeta not touching the posterior margin of the fore wing (p. 158, Figures 2 and 3 in [8]). However, the examined specimens collected from China have the clava with 8 MPS and the marginal vein with dorsal macrochaeta touching the posterior margin of the fore wing.

#### 3.2.7. *Polynema* (*Doriclytus*) *bicolorigastra* Rehmat & Anis, 2016

*Polynema* (*Doriclytus*) *bicolorigastra* Rehmat & Anis, 2016 [8], pp. 140, 144–145. 

**Figure 6:**
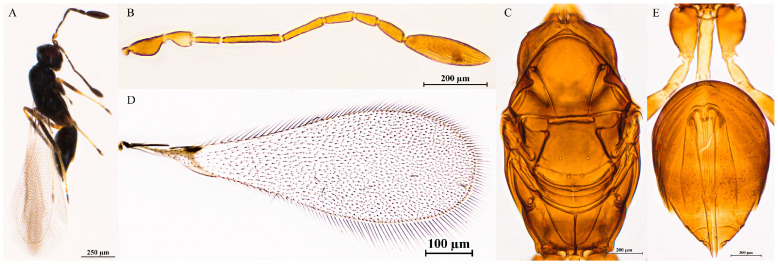
*Polynema* (*Doriclytus*) *bicolorigastra* Rehmat & Anis, 2016. female. (**A**) body; (**B**) antenna; (**C**) mesosoma in dorsal view; (**D**) fore wing; (**E**) metasoma in dorsal view.

Diagnosis. ♀ (♂ unknown). Body length 729–737 μm (n = 8; Figure 6A). Body dark brown; antenna dark brown with pedicel yellowish-brown; petiole light brown; legs dark brown with T1–T3 yellowish-brown, T4 brown. Antenna with scape smooth, 2.6–3.6× as long as wide; F2 longest funicular; F1 and F5 subequal in length; F6 with 1 MPS; clava 2.8–3.6× as long as wide, with 8 or, usually, 9 MPS (Figure 6B). Mesosoma 1.5–1.7× as long as wide; scutellum with sensilla positioned near posterior margin, scutellum with scattered foveae; propodeum with a complete median carina extending to anterior margin (Figure 6C). Forewing 3.0–3.2× as long as wide; discal setae originating behind apex of submarginal vein (Figure 6D). Ovipositor barely exserted beyond apex of gaster by 0.8–1.2× metatibia length (Figure 6E).

Material examined. CHINA: Yunnan, Honghe, 22°56′28″ N, 103°41′36″ E, 1676 m, Xu Wang: 16.IX–1.X.2022, 6♀♀; 1–15.X.2022, 1♀; 15.X–1.XI.2022, 1♀. All specimens listed above are slide-mounted and deposited in ICXU.

Distribution. Oriental region (China: Yunnan; India).

Hosts. Unknown. 

Remarks. *Polynema* (*Doriclytus*) *bicolorigastra* typically has 9 MPS on the clava, with one Chinese specimen examined here bearing 8 MPS.

#### 3.2.8. *Polynema* (*Doriclytus*) *dhenkunde* Mani & Saraswat, 1973

*Polynema dhenkunde* Mani & Saraswat, 1973 [36], pp. 116; Subba Rao & Hayat, 1986 [37], 190; Mani, 1989 [38], 1417; Hayat & Anis, 1999 [39], 323; Anis & Rehmat, 2013 [40], 7.

*Polynema* (*Doriclytus*) *dhenkunde* (Mani & Saraswat): Triapitsyn, 2013 [41], 39−41; Rehmat & Anis, 2016 [8], pp. 142–143.

**Figure 7:**
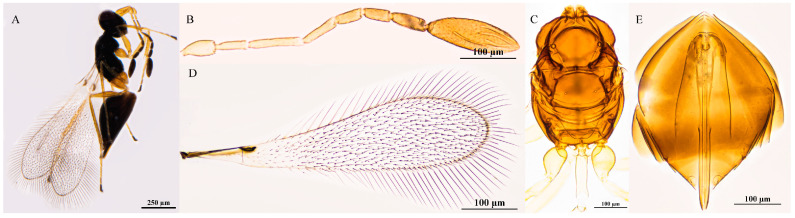
*Polynema* (*Doriclytus*) *dhenkunde* Mani & Saraswat, 1973. female. (**A**) body; (**B**) antenna; (**C**) mesosoma in dorsal view; (**D**) fore wing; (**E**) metasoma in dorsal view.

Diagnosis. ♀ (♂ unknown). Body length 514–559 μm (n = 2; Figure 7A). Body dark brown; antenna dark brown with scape and pedicel light brown; petiole light brown; legs light brown with T4 dark brown. Antenna with scape smooth, 3.0–3.6× as long as wide; F3 and F6 subequal in length, both the shortest funiculars; F6 with 1 MPS; clava 2.6× as long as wide, with 7 MPS (Figure 7B). Propodeum with a short, inconspicuous median carina (Figure 7C). Fore wing narrow, 3.7–4.3× as long as wide; discal setae extending to about mid-length of submarginal vein; marginal setae long, longest marginal seta 0.6–0.7× greatest width of fore wing (Figure 7D). Ovipositor 1.0–1.1× metatibia length (Figure 7E).

Material examined. CHINA: Henan, Zhengzhou, 34°54′20″ N, 113°39′12″ E, 65 m, 16.VIII.2024, Yanyan Liu, 1♀. Yunnan, Dali, 25°43′29″ N, 100°05′36″ E, 2924 m, 16.VIII.2024, Xiaolei Huang, 1♀. All specimens listed above are slide-mounted and deposited in ICXU.

Distribution. Oriental region (China: Henan, Yunnan; India).

Hosts. Unknown. 

Remarks. *Polynema* (*Doriclytus*) *dhenkunde* was originally described by Rehmat & Anis [8] as having F1 and F4 equal in length, whereas in the Chinese specimens studied here, F1 is longer than F4.

#### 3.2.9. *Polynema* (*Doriclytus*) *dunense* Hayat & Anis, 1999

*Polynema dunense* Hayat & Anis, 1999 [39], pp. 316, 319–320.

*Polynema* (*Doriclytus*) *dunense* (Hayat & Anis): Rehmat & Anis, 2016 [8], pp. 145.

**Figure 8:**
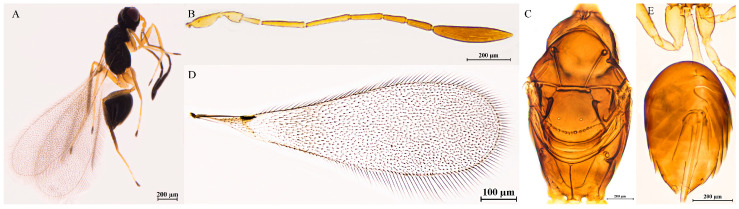
*Polynema* (*Doriclytus*) *dunense* Hayat & Anis, 1999. female. (**A**) body; (**B**) antenna; (**C**) mesosoma in dorsal view; (**D**) fore wing; (**E**) metasoma in dorsal view.

Diagnosis. ♀ (♂ unknown). Body length 756–779 μm (n = 6; Figure 8A). Body dark brown; antenna dark brown with scape and pedicel yellowish-brown; petiole yellowish-brown; legs light brown with T1–T3 yellowish-brown. Antenna with scape smooth, 3.1–3.6× as long as wide; F1 the shortest funicular; F2 and F3 subequal in length, both the longest funiculars; F6 with 1 MPS; clava relatively narrow and long, 3.8–4.9× as long as wide, with 9 MPS (Figure 8B). Mesosoma 1.6–1.7× as long as wide; scutellum with a row of frenal foveae; propodeum with a complete median carina extending to the anterior margin (Figure 8C). Fore wing 3.3–3.4× as long as wide, discal setae extend basally to about midlength of submarginal vein (Figure 8D). Ovipositor slightly exserted beyond gastral apex, ovipositor 0.6–0.7× metatibia length (Figure 8E). 

Material examined. CHINA: Yunnan, Dali, 25°43′29″ N, 100°05′36″ E, 2924 m, Xiaolei Huang: 10.VI–3.VII.2022, 4♀♀; 16.VI–2.VII.2023, 2♀♀. All specimens listed above are slide-mounted and deposited in ICXU.

Distribution. Oriental region (China: Yunnan; India).

Hosts. Unknown. 

Remarks. *Polynema* (*Doriclytus*) *dunense* was originally described by Rehmat and Anis [8] from India as having an ovipositor and a gaster subequal in length (p. 160, Figure 21 in [8]), whereas the ovipositor is significantly shorter than the gaster in Chinese specimens studied here.

#### 3.2.10. *Polynema* (*Doriclytus*) *tyakshiense* Irfan & Anis, 2023

*Polynema* (*Doriclytus*) *tyakshiense* Irfan & Anis, 2023 [7], pp. 65–66.

**Figure 9:**
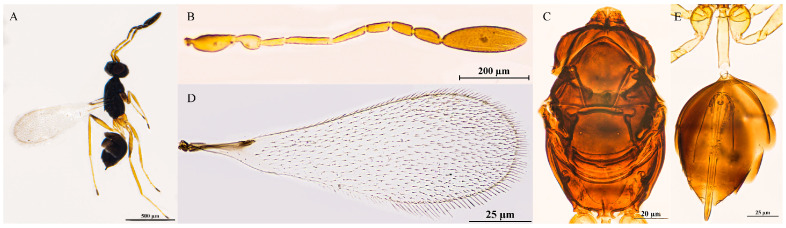
*Polynema* (*Doriclytus*) *tyakshiense* Irfan & Anis, 2023. female. (**A**) body; (**B**) antenna; (**C**) mesosoma in dorsal view; (**D**) fore wing; (**E**) metasoma in dorsal view.

Diagnosis. ♀ (♂ unknown). Body length 469–613 μm (n = 8; Figure 9A). Body dark brown; antenna brown with scape, pedicel, and F1 yellowish-brown; petiole yellow; legs yellow; T4 brown. Antenna with scape smooth, 2.7–3.4× as long as wide; F1 and F6 subequal in length; F6 with 1 MPS; clava 2.5–4.1× as long as wide, with 8 MPS (Figure 9B). Mesosoma 1.5–1.6 × as long as wide; scutellum with placoid sensilla centrally positioned, without a row of frenal foveae; propodeum with an incomplete median carina not extending to the anterior margin (Figure 9C). Fore wing 3.0–3.5× as long as wide; marginal setae short, longest marginal seta 0.2–0.4× greatest width of fore wing (Figure 9D). Ovipositor 0.9–1.0× metatibia length (Figure 9E).

Material examined. CHINA: Guangxi: Guigang, 23°11′04″ N, 109°31′55″ E, 277 m, 19.VIII.2024, Huayan Chen, 2♀. Laibin, 22°44′28″ N, 109°16′36″ E, 63 m, 19.VIII.2024, Zelu Mu, 1♀. Guizhou, Anshun, 25°44′52″ N, 106°05′00″ E, 1150 m, 15.VIII.2024, Zelu Mu, 1♀. Hunan: Chenzhou, 24°56′44″ N, 112°54′08″ E, 1108 m, 18–28.II.2021, Xiaolong Lin, 1♀. Shanghai, 31°13′43″ N, 121°28′29″ E, 75 m, 1.VIII.2023, Xiaolong Lin, 1♀. Xizang, Rikaze, 27°28′57″ N, 88°54′27″ E, 2832 m, 20.VII.2021, Qingtao Wu, 2♀♀. All specimens listed above are slide-mounted and deposited in ICXU.

Distribution. Oriental region (China: Guangxi, Guizhou, Hunan, Xizang; India).

Hosts. Unknown. 

Remarks. *Polynema* (*Doriclytus*) *tyakshiense* was originally described by Irfan & Anis [7] from India as having the clava with 6 MPS (p. 66, Figure 3 in [7]), scutellum with a row of frenal foveae (p. 66, Figure 8 in [7]), and a petiole only slightly longer than the metacoxa (p. 66, Figure 9 in [7]). However, the Chinese specimens studied here have the clava with 8 MPS, scutellum without a row of frenal foveae, and a petiole significantly longer than the metacoxa.

## 4. Discussion

This integrative taxonomic study elucidated the diversity of *Polynema* (*Doriclytus*) in the Oriental region of China, addressing the long-standing challenges posed by minute body size and high morphological similarity in this group. The remarkable concordance among morphological classifications, ASAP partitioning, and GMYC delimitation revealed three new species to science and five new species records for China.

Genetic analysis revealed a clear barcoding gap, with maximum intraspecific divergence of 1.51% and interspecific divergences ranging from 3% to 12% (Supplementary Material, Table S1) within the 470 bp COI barcode region. This pattern, consistent with typical mymarid species boundaries, was independently supported by both molecular delimitation methods. The ASAP analysis successfully partitioned sequences into species groups that matched morphological identifications, while the GMYC model effectively distinguished between inter- and intraspecific branching patterns. This congruence between distance-based and tree-based approaches, despite their different methodological assumptions, provides robust validation of our species hypotheses and demonstrates the effectiveness of integrative taxonomy in resolving boundaries within morphologically conserved groups.

We acknowledge that species delimitation based solely on the mitochondrial COI marker has inherent limitations, including potential confounding effects from incomplete lineage sorting and nuclear mitochondrial pseudogenes. However, the robustness of our conclusions is supported by multiple lines of evidence: (1) rigorous quality control through bidirectional sequencing and amino acid translation minimized pseudogene contamination; (2) strong congruence between molecular (ASAP/GMYC) and morphological delimitations; and (3) a clear barcoding gap with intraspecific distances <2% versus interspecific distances >3%. Collectively, these cross-validations demonstrate the reliability of our species delimitations despite the theoretical constraints of single-locus data.

Prior to this study, molecular resources for *Polynema* systematics were compromised by numerous unverified sequences in GenBank. Our deposition of 32 novel COI sequences with morphologically verified vouchers significantly enriches these resources, providing reliable references for rapid identification and establishing a foundation for broader phylogenetic investigations. The discovery of three new species from limited sampling efforts, combined with the five new records, substantially expands knowledge of *P.* (*Doriclytus*) diversity in Oriental China. Given the region’s complex topography and diverse climatic zones remaining largely unexplored for these minute parasitoids, our findings likely represent only a fraction of the actual diversity, highlighting the need for expanded sampling and continued integrative taxonomic efforts in this economically important group of egg parasitoids.

## Data Availability

All DNA sequences generated in this study have been deposited in the GenBank database under accession numbers PX108759-PX108790. Type and voucher specimens are deposited in the Insect Collection of the College of Life Science and Technology, Xinjiang University, Urumqi, China (ICXU) and are available for examination upon reasonable request to the corresponding author.

## Supplementary Materials

The following supporting information can be downloaded at: https://www.mdpi.com/article/10.3390/insects16111166/s1, Figure S1: GMYC species delimitation of *Polynema* (*Doriclytus*) based on COI sequences; Table S1: Kimura 2-parameter (K2P) intra- and interspecific pairwise distances. References [23,42] are cited in the Supplementary Materials.
