# Supplementary figures and images for: Integrative Taxonomy of Polynema (Doriclytus) (Hymenoptera: Mymaridae) from Oriental China: Three New Species and Five New Records Revealed by Morphological and Molecular Analyses
Source: Insects. 2025 Nov 15;16(11):1166. doi: 10.3390/insects16111166 (PMC12653764; doi:10.3390/insects16111166)

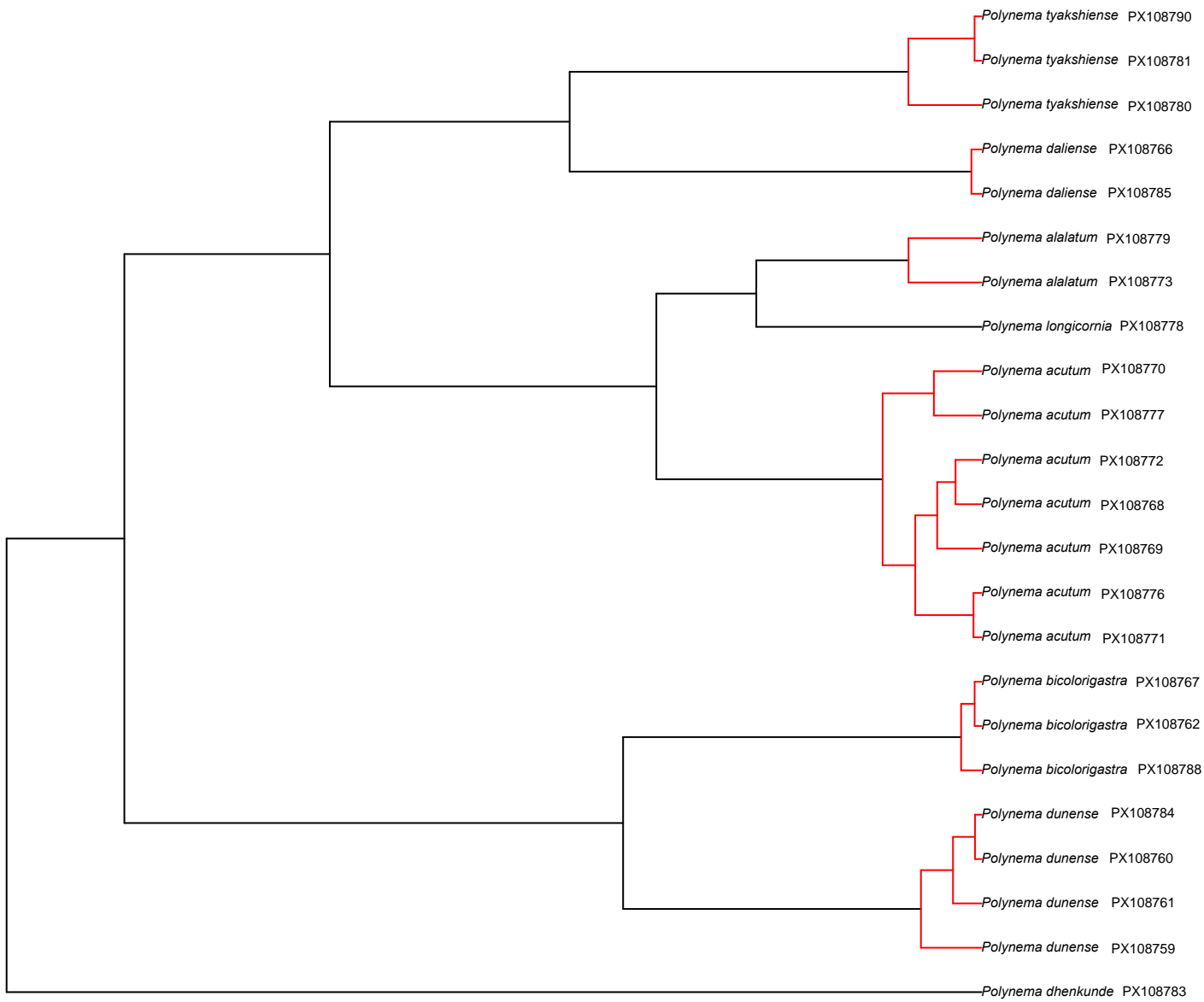

Supplement: Supplementary file 1 [file insects-16-01166-s001.zip › insects-3918768-Figure S1. GMYC Species delimitation of Polynema (Doriclytus) based on COI sequences.pdf]
